# The Free and Cued Selective Reminding Test Predicts Braak Stage

**DOI:** 10.3233/JAD-200980

**Published:** 2021-03-09

**Authors:** Ellen Grober, Qi Qi, Lynn Kuo, Jason Hassenstab, Richard J. Perrin, Richard B. Lipton

**Affiliations:** a Department of Neurology, Albert Einstein College of Medicine and Montefiore Medical Center, Bronx, NY, USA; b Department of Statistics, University of Connecticut, Storrs, CT, USA; c Department of Neurology, Washington University School of Medicine, St. Louis, MO, USA; d Department of Pathology and Immunology, Washington University School of Medicine, St. Louis, MO, USA

**Keywords:** Alzheimer’s disease, braak stage, clinical dementia rating scale –sum of boxes, free and cued selective reminding test, mini-mental state exam, neuropathology

## Abstract

**Background:**

The ultimate validation of a clinical marker for Alzheimer’s disease (AD) is its association with AD neuropathology.

**Objective:**

To identify clinical measures that predict pathology, we evaluated the relationships of the picture version of the Free and Cued Selective Reminding Test (pFCSRT + IR), the Mini-Mental State Exam (MMSE), and the Clinical Dementia Rating scale Sum of Boxes (CDR-SB) to Braak stage.

**Methods:**

315 cases from the clinicopathologic series at the Knight Alzheimer’s Disease Research Center were classified according to Braak stage. Boxplots of each predictor were compared to identify the earliest stage at which decline was observed and ordinal logistic regression was used to predict Braak stage.

**Results:**

Looking at the assessment closest to death, free recall scores were lower in individuals at Braak stage III versus Braak stages 0 and I (combined) while MMSE and CDR scores for individuals did not differ from Braak stages 0/I until Braak stage IV. The sum of free recall and total recall scores independently predicted Braak stage and had higher predictive validity than MMSE and CDR-SB in models including all three.

**Conclusion:**

pFCSRT + IR scores may be more sensitive to early pathological changes than either the CDR-SB or the MMSE.

## INTRODUCTION

The ultimate test of a clinical marker for Alzhei-mer’s disease (AD) is its association with AD neuropathology. The clinical marker in this report is performance on the picture version of Free and Cued Selective Reminding Test (pFCSRT + IR) with Immediate Recall [1, 2]. Unlike other episodic memory tests, the FCSRT begins with an encoding phase in which participants identify items (e.g., grapes) in response to category cues (fruit). Controlling cognition in this way assures that participants engage in the type of semantic processing that maximizes learning [3]. These same category cues are then used in the test phase to prompt recall of items not retrieved by free recall. Control of cognitive processing permits measurement of retrieval impairment defined by free recall (FR) separately from storage impairment defined by cued recall. The FCSRT is a well-established test in research and clinical settings. Since 2007, performance on the FCSRT has been used by the International Workgroup (IWG) to define memory impairment, the core clinical phenotype of AD [4, 5].

Our objective was to examine the association between pFCSRT + IR performance and neurofibrillary tangle (NFT) pathology. Previously, in 28 cases on the AD continuum [6], we examined the relationship between free recall and stages in the distribution of NFT pathology in AD defined by Braak and Braak [7]. Looking at the assessment closest to death, free recall scores were lower in individuals at Braak stage III versus Braak stage II whereas mental status, defined by the Blessed Information Memory Concentration test (BIMC) [8], was not lower until Braak stage IV, suggesting that free recall may serve as a cognitive marker of AD at an earlier neuropathologic stage than mental status.

We sought to replicate and extend these findings using the longitudinal clinicopathologic series from the Charles F. and Joanne Knight ADRC at Washington University in St. Louis [9]. We compared three different approaches for assessing the severity of AD: a clinical staging method, a mental status approach, and an approach based on memory testing. As this was a retrospective study linked to autopsy, we were limited to the measures collected in life. The Clinical Dementia Rating (CDR) Scale developed at Washington University nearly 40 years ago has become the standard clinical staging system for the Alzheimer’s disease continuum [10, 11]; the Mini-Mental State Exam (MMSE) has been the standard measure of overall cognitive functioning/mental status for more than 40 years [12]; and the pFCSRT + IR developed at Einstein more than 30 years ago and recently used to define stages in the breakdown of episodic memory [13]. In this sample, all three assessments were usually obtained within a two-week period. To minimize the potential for pathologic change between cognitive assessment and death, we selected the values of these measures from their last assessments before death as the predictors.

We hypothesized that free recall impairment would be evident at an earlier Braak stage than would the MMSE or the CDR sum of boxes (CDR-SB). Total recall (TR), the sum of free and cued recall, would show decline at a more advanced Braak stage than would free recall. We also sought to determine which test best predicts the Braak stage. For the pFCSRT + IR, the combination of FR and TR has been shown to be the most effective measure in signaling decline in preclinical AD cognitive composites [14, 15]. We hypothesized that their combination would outperform the MMSE in predicting Braak stage.

Whether the combination would outperform the CDR-SB was to be determined. At the Knight ADRC, CDR-SB scores are incorporated in the rendering of research clinical diagnoses and are disclosed to the neuropathologists. The lack of independence between CDR-SB scores and neuropathology could inflate their association. Thus, an outcome in which FR + TR predicted Braak stage as effectively as CDR-SB would be considered a successful demonstration of the test’s validity.

## METHODS

### Study sample

We used clinical, cognitive, and neuropathological data from autopsied cases evaluated annually prior to death at the Knight ADRC at Washington University in St. Louis. In 2004, the pFCSRT + IR was added to the assessment. Since then, 326 autopsies were conducted on participants who performed the pFCSRT + IR at least once before their death. After eliminating participants with missing values in the demographic and clinical characteristics, we conducted analyses on 315 participants using scores from their last assessment before death. The sample size of statistical analysis with different predictors varied from 311 to 315 because of a few missing items in the predictors. Written informed consent was obtained from all participants.

### Predictors

#### pFCSRT + IR [2]

There are four versions of the pFCSRT + IR. The version used here includes pictures and immediate recall during the study phase. The test begins by having participants search a card containing four line drawings (e.g., grapes) for an item that goes with a unique category cue (e.g., fruit). After all four items are identified, immediate cued recall of just those four items is tested. The encoding phase is repeated for a total of 16 drawings. The test phase begins with a period of free recall (FR), in which a participant must list as many of the 16 items as possible, without any cues, immediately followed by cued recall for items not retrieved by FR. When cued recall fails, the participant is reminded of the item by saying, “The fruit was the grapes, what was the fruit?” There are three test trials. The sum of FR and cued recall is total recall (TR). The trajectories of FR (maximum score = 48) and TR (max = 48) were examined.

#### CDR-SB [10, 11]

The CDR was developed at the Knight ADRC and has become the standard clinical staging system for the AD continuum. The CDR-SB is a summary score of the severity of impairment from 0 (none) to 3 (severe) in each of six clinical domains: memory, orientation, judgement and problem solving, community affairs, home and hobby, and personal care. High scores indicate greater impairment (max = 18). Clinical diagnoses and CDR-SB scores are determined without reference to cognitive test scores.

#### MMSE [12]

Since its introduction, the MMSE has been used in longitudinal aging studies, clinical practice, and clinical trials as an indicator of overall cognitive functioning. It includes brief tests of orientation, memory, attention, and language (max = 30). Low scores indicate greater impairment.

### Neuropathological assessment

Details of brain autopsy and histological processing have been described in detail previously [16]. All cases were assigned a Braak NFT stage with revised methods that adapted tissue selection and processing and introduced PHF-1 immunohistochemistry for hyperphosphorylated tau protein using monoclonal antibody PHF-1.

### Statistical analyses

We performed Kruskal-Wallis rank sum tests for continuous variables to test the equivalence of means at different Braak stages. To examine the independence of categorical variables and Braak stage, we performed Chi-squared tests of independence.

To examine changes in FR, TR, CDR-SB, and MMSE as a function of Braak stage, we overlaid boxplots of each individual predictor for each Braak stage. We conducted Kruskal-Wallis rank sum tests and pairwise comparisons for different Braak stages of each predictor using Dunn tests [17] to identify the earliest stage at which decline was observed.

Ordinal logistic regression (proportional odds) was used to predict the Braak stage using FR + TR, MMSE, and CDR-SB in separate models and then combined in a single model [18]. Each analysis modeled the logit transformations of the ordered Braak probabilities using simultaneous linear equations sharing the same slope coefficients. The method makes the parallel regression assumption for all variables across the levels of Braak stage which was validated using the Brant test before running the regression models [19]. Details of the Brant test are provided in the Supplementary Material.

We first ran models including each cognitive assessment and the covariates one at a time and then ran a final full model, including all three cognitive tests and covariates that were significant in earlier models. We used delta pseudo R^2^ to measure the incremental explanatory power of each of the predictors, FR + TR, MMSE, and CDR-SB. The delta pseudo R^2^ of each predictor is the difference of pseudo R^2^ between the full model and the model excluding each predictor [18, 20]. We also conducted a likelihood ratio test (LRT) between each reduced model and the full model to examine the incremental explanatory power of each predictor.

## RESULTS

The eligible sample for this study included 315 participants who performed the pFCSRT + IR at least once before their death. There were only four participants at Braak stage 0. Because of the limited sample size, we combined Braak stages 0 and I. The demographic and clinical characteristics of the sample classified by Braak stage are shown in Table 1. Age differed among the different Braak stages with the oldest individuals being in Braak stages II-V (*p* < 0.0001); the lag time from assessment to death, and the proportion of *APOE* ɛ4 genotype (presence versus absence of an ɛ4 allele) increased significantly across Braak stages (*p* < 0.001). Education and sex (the proportion of women) did not differ significantly by Braak stage.

**Table 1:** Characteristics of Participants Classified by Braak NFT Stage

	N	Age	Sex	Education	*APOE* ɛ4	Lag from test to death
Braak stage		Mean (SD)	%Female	Mean (SD)	%	Mean (SD)
0/I	46	82.30 (10.72)	39.13	14.87 (2.78)	15.22	2.58 (2.45)
II	45	88.20 (7.32)	55.56	15.47 (3.10)	26.67	2.44 (2.16)
III	33	88.42 (10.07)	45.45	14.91 (3.13)	27.27	2.58 (2.17)
IV	27	88.63 (7.70)	59.26	14.52 (3.79)	29.63	2.64 (1.97)
V	118	87.03 (7.13)	54.24	14.36 (3.01)	56.78	4.10 (2.57)
VI	46	80.15 (8.93)	36.96	14.39 (3.08)	76.09	5.53 (3.07)
Total	315	85.78 (8.88)	49.21	14.67 (3.09)	43.81	3.56 (2.70)
p		< 0.0001	0.166	0.588	< 0.0001	< 0.0001

### Comparison of adjacent Braak stages

**Fig. 1:**
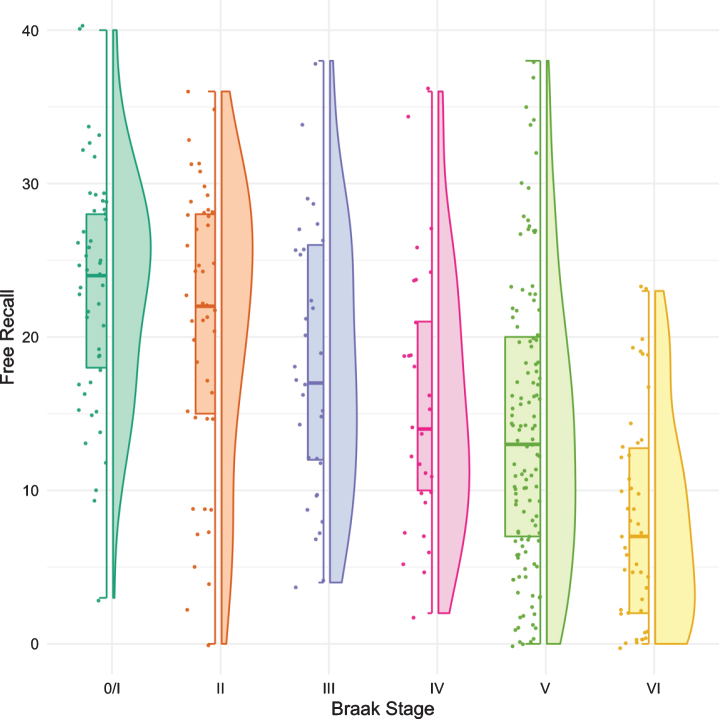
Boxplots and violin plots for free recall as a function of Braak stage.

Figure 1 shows boxplots and violin plots of free recall score by Braak stage. The line near the center of each box represents the median free recall score in the Braak category. The top of each box represents the 75th percentile while the bottom represents the 25th percentile. The width of each box represents the number of cases in a particular Braak stage. Higher FR scores represent better memory. The *p*-values for pairwise comparisons of the FR scores between any Braak stages are shown in Table 2. FR at Braak II does not differ from Braak 0/I. FR at Braak III is significantly lower than FR at Braak 0/I but does not differ from Braak II. FR at Braak IV is significantly lower than at Braak stage II and earlier stages. FR at Braak V is significantly lower than at Braak stage III and earlier stages. FR at Braak VI is significantly different from all others.

**Table 2:** *p*-values from the Pairwise Comparison of FR by Braak Stages

	Braak	Braak	Braak	Braak	Braak	Braak
	0/I	II	III	IV	V	VI
Braak 0/I	-	0.280	0.028	0.002	< 0.001	< 0.001
Braak II		-	0.255	0.031	< 0.001	< 0.001
Braak III			-	0.325	0.031	< 0.001
Braak IV				-	0.330	0.002
Braak V					-	0.002

**Fig. 2:**
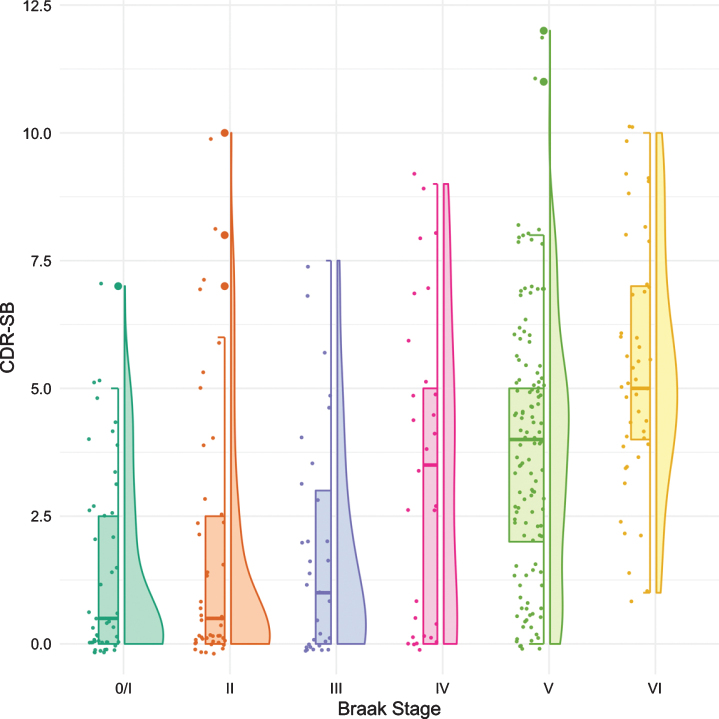
Boxplots and violin plots for CDR-SB as a function of Braak stage.

Figure 2 shows the boxplots and violin plots for the CDR-SB. Higher CDR-SB scores represent greater impairment. Performance was similar from Braak stages 0/I to stage III. The first “decline” was not seen until Braak stage IV. The next “decline” was observed at Braak stage VI. The *p*-values for pairwise comparisons of the CDR-SB between any Braak stages are shown in Table 3.

**Table 3:** *p*-values from the Pairwise Comparison of CDR-SB by Braak Stages

	Braak	Braak	Braak	Braak	Braak	Braak
	0/I	II	III	IV	V	VI
Braak 0/I	-	0.776	0.656	0.004	< 0.001	< 0.001
Braak II		-	0.805	0.009	< 0.001	< 0.001
Braak III			-	0.027	< 0.001	< 0.001
Braak IV				-	0.335	0.003
Braak V					-	0.004

**Fig. 3:**
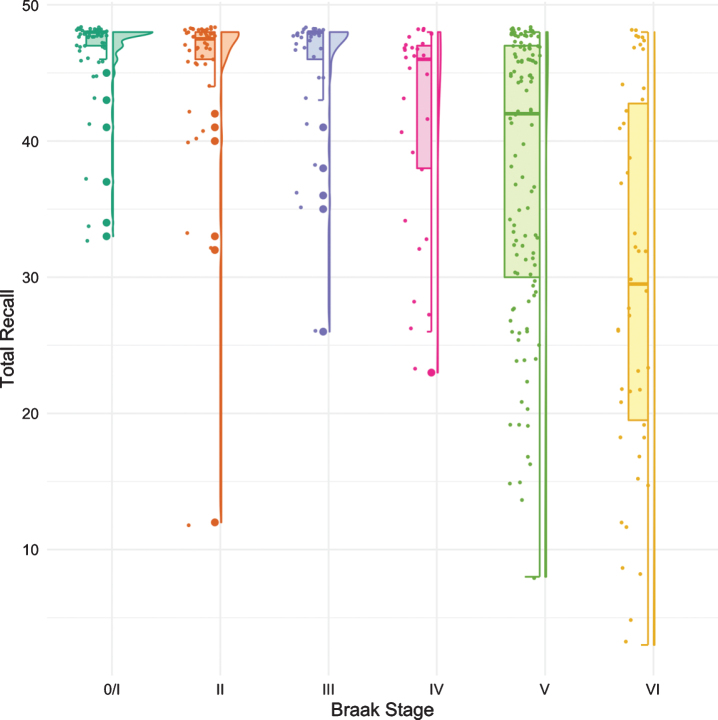
Boxplots and violin plots for TR as a function of Braak stage.

**Fig. 4:**
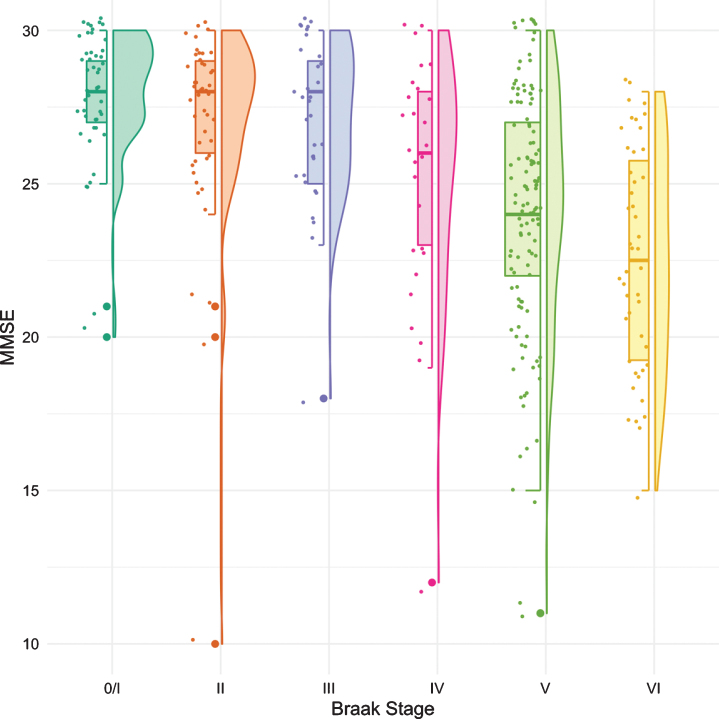
Boxplots and violin plots for MMSE as a function of Braak stage.

The same methodology was used to assess differences in test performance between Braak stages for TR and for MMSE. While FR provided a signal of early stage NFT pathology, TR was not significantly different from Braak 0/I until Braak stage IV (Fig. 3, Table 4). The first “decline” in MMSE, like the CDR + SB, was not observed until Braak stage IV followed by another “decline” at Braak stage VI (Fig. 4, Table 5).

**Table 4:** *p*-values from the Pairwise Comparison of Total Recall by Braak Stages

	Braak	Braak	Braak	Braak	Braak	Braak
	0/I	II	III	IV	V	VI
Braak 0/I	-	0.449	0.642	0.002	< 0.001	< 0.001
Braak II		-	0.797	0.017	< 0.001	< 0.001
Braak III			-	0.016	< 0.001	< 0.001
Braak IV				-	0.316	0.003
Braak V					-	0.006

**Table 5:** *p*-values from the Pairwise Comparison of MMSE by Braak Stages

	Braak	Braak	Braak	Braak	Braak	Braak
	0/I	II	III	IV	V	VI
Braak 0/I	-	0.208	0.222	0.002	< 0.001	< 0.001
Braak II		-	0.906	0.051	< 0.001	< 0.001
Braak III			-	0.084	< 0.001	< 0.001
Braak IV				-	0.196	0.007
Braak V					-	0.034

**Fig. 5:**
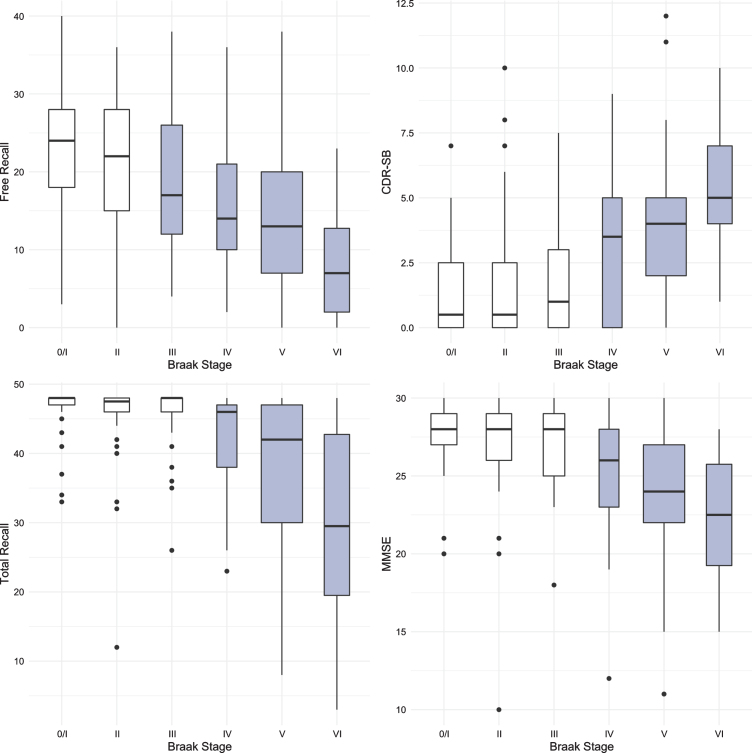
Boxplots for FR, TR, MMSE, and CDR-SB with the earliest Braak stage at which a score was distinguishable from the score at Braak stage 0/I shown in grey.

The results for all four predictors are summarized in Fig. 5. The earliest Braak stage at which a score was distinguishable from the score at Braak stage 0/I is shown in grey. FR generated a signal at Braak stage III, before the first signals generated by TR, MMSE and CDR-SB at Braak stage IV.

### Do cognitive assessments at the time closest to death predict Braak stage?

The results of the Brant test for checking the proportional odds assumption of the ordinal logit models are included in the Supplementary Material (Supplementary Table 1). Table 6 shows the results of the models for predicting Braak stages using FR + TR (Model 1 prime), MMSE (Model 2 prime), and CDR-SB (Model 3 prime) separately. Neither education nor sex were significant risk factors in any model. In all models, cases with any *APOE* ɛ4 allele were three times as likely to have a higher Braak stage than cases without. In Models 1 prime and 2 prime, for each yearly increase in time between last assessment and death, the odds of a higher Braak stage increased to 1.2 times. This covariate was eliminated in the CDR-SB (Model 3 prime) because it violated the proportional odds assumption. With regard to the cognitive predictors, participants with a 1-point increase in FR+TR were 5%less likely to have a higher Braak stage; cases with 1-point increase in MMSE were 18%less likely to have a higher Braak stage and participants with 1-point increase in CDR-SB were 36%more likely to have a higher Braak stage.

**Table 6:** Ordinal Logistic Regression Results for predicting Braak stage using FR + TR (Model 1 prime), MMSE (Model 2 prime), CDR-SB (Model 3 prime) and the full model (Model 4) as predictors, adjusting for demographic covariates including time to death, education, *APOE*, and sex as permitted by the Brant test

	Model 1 prime			Model 2 prime			Model 3 prime			Model 4		
	Estimate (SE)	OR	p	Estimate (SE)	OR	p	Estimate (SE)	OR	p	Delta pseudo *R*^2^	OR	p
FR + TR	–0.048 (0.007)	0.953	< 0.001							0.0222	0.963	< 0.001
MMSE				–0.193 (0.031)	0.824	< 0.001				0.0029	0.935	0.086
CDR-SB							0.308 (0.044)	1.361	< 0.001	0.0034	1.112	0.064
Time to death	0.179 (0.045)	1.196	< 0.001	0.244 (0.044)	1.276	< 0.001
EDUCclass > = 16	0.063 (0.239)	1.065	0.793	0.133 (0.238)	1.142	0.577	0.016 (0.240)	1.016	0.948
EDUCclass 13–15	0.014 (0.296)	1.014	0.963	–0.005 (0.296)	0.995	0.986	–0.076 (0.295)	0.927	0.795
*APOE* ɛ4	1.062 (0.234)	2.892	< 0.001	1.107 (0.234)	3.025	< 0.001	1.201 (0.232)	3.323	< 0.001	0.0194	2.829	< 0.001
Sex							0.167 (0.214)	1.182	0.436

Model 1 prime: Braak stage on FR + TR adjusting for time to death, education, and *APOE*, sample size is 314; Model 2 prime: Braak stage on MMSE adjusting for time to death, education, and *APOE*, sample size is 312; Model 3 prime: Braak stage on CDR-SB adjusting for education, *APOE*, and sex, sample size is 315; Model 4: Braak stage on FR + TR, MMSE, and CDR-SB adjusting for significant covariates (*APOE*), sample size is 311.

The full model that includes all predictors controlling for the significant covariate of *APOE* ɛ4 genotype is shown in Table 6 (Model 4). The odds ratio for FR + TR was nearly identical to when it was the only predictor (Model 1 prime). FR + TR was still significant (*p*-values < 0.001) while MMSE and CDR-SB fell just short of statistical significance (Model 4). The improvement in model fit (delta pseudo *R*^2^) was higher for FR + TR (0.0222) than for CDR-SB (0.0034) or MMSE (0.0029). The odds ratio of *APOE* *ɛ*4 was similar to the models with each predictor separately. FR + TR enhanced the explanatory power significantly when CDR-SB and MMSE were in the model (*p* < 0.001) while CDR-SB was short of significance when the other two predictors were in the model (*p* = 0.086 and *p* = 0.064).

## DISCUSSION

We sought to determine whether episodic memory assessed with the pFCSRT + IR was a better predictor of Braak stage than mental status assessed with the MMSE or overall clinical functioning assessed with the CDR-SB. ‘Better’ was defined in two ways: the measure that provided the earliest signal of NFT pathology and the measure that best predicted Braak stage.

The cohort consisted of 315 well characterized cases from the clinicopathologic series at the Knight ADRC classified according to Braak NFT stage. Free recall was the measure that provided the earliest signal of NFT pathology shown by significantly lower score at Braak III compared to the score at Braak 0/I, essentially replicating our earlier findings [6]. It was not until NFT pathology reached Braak IV that a significant difference from Braak stage 0/I could be detected for either the MMSE or the CDR-SB. A significant difference was also observed at Braak IV for TR when the score fell below 32, indicating a moderately severe memory impairment [21].

FR + TR outperformed CDR-SB, and MMSE in predicting Braak stage. All three were significant predictors in separate ordinal logistic regression models but when combined, FR + TR remained significant whereas MMSE and the CDR-SB lost statistical significance. The presence of an *APOE* ɛ4 allele increased the odds of having greater NFT pathology to a similar extent in all the ordinal logistic regression models. The covariates of age, education, sex, and lag time were not included in the full model because they violated the proportional odds assumption of the ordinal logit model. In the full model that included the *APOE* ɛ4 covariate, FR + TR had greater explanatory power than that of the CDR-SB and the MMSE and enhanced the explanatory power of the model significantly in the presence of the others.

The early emergence of FR impairment on the pFCSRT + IR + IR in the predementia phase of AD has been well documented in clinical studies [22–25]. Dementia-free community volunteers from the Einstein Aging Study (EAS) who displayed impaired FR at baseline (< = 24) developed dementia at dramatically higher rates over five years than participants with intact FR [26]. Decline of FR was shown to occur even earlier by aligning incident AD cases from the Baltimore Longitudinal Study of Aging (BLSA) on time of diagnosis and then examining FR over the preceding years [27]. Approximately seven years before diagnosis, the rate of FR decline accelerated from intact levels (> 30) with a second acceleration 2 to 3 years before diagnosis (< = 24). This trajectory was replicated in a larger BLSA cohort [28].

pFCSRT + IR measures, FR, TR, and their combination are components in the preclinical Alzheimer’s disease clinical composite (PACC) for detecting cognitive change which also includes Logical Memory, Digit Symbol Substitution Test, and the MMSE [29, 30]. All combinations including FR resulted in larger effect sizes for differences between clinically normal participants grouped according to threshold levels of amyloid imaging over three and five years of follow-up [14]. FR alone or combined with total recall were the only individual components to show differences between the Aβ  + group who progressed to CDR 0.5 versus those that remained stable.

The failure of clinical trials targeted to decreasing the accumulation of Aβ pathology in cognitively normal adults prompted the search for any change in the PACC that occurs within the normal range of the amyloid tracer, ^18^F-florbetapir [15]. When continuous levels of the tracer were associated with individual PACC components, the magnitude of the decrease in FR and FR + TR scores at subclinical levels of tracer uptake compared to normal levels was more than twice that of the other PACC components with a larger magnitude of effect than the PACC itself. Though the decline in pFCSRT + IR performance in the subthreshold range of Aβ was small, it marks the start of episodic memory impairment that is the hallmark of AD.

A limitation of the current study was the inclusion of all autopsy cases with pFCSRT+IR data without regard to the presence of non-AD neuropathologies. Most cases had some degree of vascular neuropathology and approximately 30%of the cohort had one or more non-AD pathologies (e.g., forms of frontotemporal lobar degeneration, Lewy body pathology, hippocampal sclerosis of aging). We cannot exclude that non-AD neuropathologies in some of these cases may have contributed to or been responsible for low FR and TR scores, because many such comorbidities can impact memory functioning [31]. Another limitation was that the demographic covariates of age, education, sex, and lag time were not included in the full ordinal logit model in order to meet the proportional odds assumption, raising the risk of unadjusted confounders.

A limitation of all clinical-pathologic correlation studies is the lag time between time of death when neuropathology is assessed and the point in time closest to death when the predictors are assessed. To address variation from person to person in the time from *in vivo* assessment to death, we included a lag variable in our models though lag variables may not fully resolve the issue. For example, participants with severe AD neuropathology (Braak stages V and VI) had the longest lag times from last assessment to death, most likely because the severity of their cognitive impairment precluded assessment. However, the lag from ascertainment of predictor variables to death in this study was uniform within person across predictor variables; as a consequence differences among predictors is unlikely to be related to differences in the interval from assessment to death. Even this assumption can be challenged if the rate of change in the predictor varies from predictor to predictor at various pathologic stages. An alternative approach is to use longitudinal data on clinical and cognitive measures as exemplified by Wilson and colleagues [31]. This approach requires additional exploration.

It is important to remember that the findings were achieved with the version of the FCSRT that uses pictures and includes immediate recall during the encoding phase. The scores from the versions that uses words without immediate recall are not equivalent to the pFCSRT + IR [32].

## CONCLUSION

Impairments in FR were associated with early NFT burden. The sum of FR and TR scores outperformed MMSE and CDR-SB in predicting Braak stage. pFCSRT + IR performance may be useful in predicting tau positivity in observational studies and in clinical trials.

## Supplementary Material

Supplementary Material
