# Supplementary material for: The Free and Cued Selective Reminding Test Predicts Braak Stage
Source: J Alzheimers Dis. 2021 Mar 9;80(1):175–83. doi: 10.3233/JAD-200980 (PMC8075386; doi:10.3233/JAD-200980)
Supplement: Supplementary Material [file jad-80-jad200980-s001.pdf]

Supplementary Material

The Free and Cued Selective Reminding Test Predicts Braak Stage

Ordinal logistic regression (proportional odds) was used to predict the Braak stage using FR+TR, MMSE, and CDR-SB in separate models and then combined in a single model [1]. Each analysis modeled the logit transformations of the ordered Braak probabilities using simultaneous linear equations sharing the same slope coefficients.

$$logit(\Pr(Y > j)) = \log\left(\frac{\Pr(Y > j)}{\Pr(Y \leq j)}\right) = \eta_j + \sum_k \beta_k\, x_k, j = 1, \dots, J - 1$$

where Y is the value of a Braak stage, J is the number of possible Braak stages, η_j is the intercept for the jth stage, x_k is the kth predictor (FR+TR for example) or covariate (age, time from last assessment to death, education, *APOE* ε4 genotype, and sex) and β_k is the corresponding coefficient. Ordinal logit regression is a proportional odds model, where the odds ratio of making response $Y > j$ at $x_k = x_1$ versus $x_k = x_2$ is $\exp(\beta_k(\mathrm{x}_1 - \mathrm{x}_2))$ which is independent of the choice of category (j). If x_k is a continuous variable, a significantly positive β_k , i.e., $\exp(\beta_\mathrm{k}) > 1$, indicates increase in x_k leads to a larger $\Pr(Y > j)$, i.e., associates with a higher stage of Y; for a categorical variable x_k, a significantly positive β_k means a specific level of x_k associates with a higher stage of Y relative to the reference level of x_k.

We conducted the Brant test [2] for checking the proportional odds assumption of the ordinal logit model for predicting Braak stage using FR+TR (Model 1), MMSE (Model 2), and CDR-SB (Model 3). p values from the Brant test are shown in Supplementary Table 1. For all three models, significant results (p<0.001) show the violation of the proportional odds assumption. We removed the most significant covariate from each model and examined the updated model with

the Brant test. If the updated model still violated the proportional odds assumption, we would eliminate the most significant covariate from the updated model until we obtain a model with a nonsignificant result. Therefore, for Model 1 and Model 2, we excluded age and sex to obtain Model 1 prime and Model 2 prime which satisfied the proportional odds assumption. We eliminated age and time to death from Model 3 to obtain Model 3 prime and the proportional odds assumption held. The Brant test for the full model that includes all predictors of Braak stage controlling for the significant covariate of *APOE* ε4 genotype which satisfied the proportional odds assumption is shown in Table (Model 4).

REFERENCES

[1] McFadden D (1973) Conditional logit analysis of qualitative choice behavior In *Frontiers in Econometrics*, Zarembke P, ed. Academic Press, New York, pp. 105-142.

[2] Brant R (1990) Assessing proportionality in the proportional odds model for ordinal logistic regression. *Biometrics*, 1171-1178.

Supplementary Table 1. p-values from the Brant Test for Checking the Proportional Odds Assumption of Ordinal Logit Models.

	Model 1	Model 1 prime	Model 2	Model 2 prime	Model 3	Model 3 prime	Model 4
Omnibus Test	<0.001	0.344	<0.001	0.128	<0.001	0.178	0.695
FR+TR	0.418	0.518					0.852
MMSE			0.033	0.076			0.292
CDR-SB					0.340	0.305	0.429
Age	0.003		0.001		0.002		
Time to death	0.006	0.092	0.005	0.093	0.001		
Education	0.264	0.303	0.316	0.265	0.430	0.323	
APOE ε4	0.543	0.798	0.587	0.827	0.573	0.808	0.706
Sex	0.069		0.104		0.096	0.030	

Note: First row reports p-values for the Omnibus Test assuming the parallel regression assumption holds. The other entries are the type III p-values for each predictor (covariate) given other covariates (covariates and predictor) in the model.
